# Dataset of endodontic microorganisms killed at 265 nm wavelength by an ultraviolet C light emitting diode in root canals of extracted, instrumented teeth

**DOI:** 10.1016/j.dib.2021.107750

**Published:** 2021-12-23

**Authors:** Kimberly A. Morio, Robert H. Sternowski, Kim A. Brogden

**Affiliations:** a Apex Endodontics, Hiawatha, IA United States; b Softronics, Ltd., Marion, IA United States; c College of Dentistry, Iowa Institute for Oral Health Research, the University of Iowa, Iowa City, IA United States

**Keywords:** Ultraviolet C, UVC, Light emitting diode, LED, Antimicrobial, Root canal infection, Endodontic infection

## Abstract

Ultraviolet C (UVC) light emitting diode (LED) can kill the endodontic pathogen *Enterococcus faecalis* and has the potential to kill other oral microorganisms associated with endodontic infections. This same bacteriocidal device shows great promise in the stimulation of periapical healing and pain reduction resulting from inflammation in root canals. Previously, we found that 255 nm UVC LED killed *E. faecalis* and induced the production of cellular biomarkers in HEPM cells and gingival fibroblasts (Morio et al., 2019). Here, we extend those findings and hypothesize that UVC LED at other wavelengths and power levels kill microorganisms associated with root canal infections. Units emitting UVC LED at 265 nm (12 mW), 265 nm (22.5 mW), and 280 nm (8 mW) wavelenths were assembled and the energy levels of their emissions were measured. The energy doses in millijoules (mJ) were calculated from the power readings of the meter (µW) × time of exposure (seconds). *Ex vivo* models of root canals were prepared in extracted, instrumented, single canal human premolars. Five cultures of microorganisms were treated with 265 nm (12 mW), 265 nm (22.5 mW), or 280 nm (8 mW) UVC LED on discs in laboratory assays and 4 cultures of microorganisms were treated with 265 nm (22.5 mW) UVC LED in root canals of extracted, instrumented teeth. After UVC LED treatment, all microorganisms were cultivated on microbiological media. Colony forming units (CFU) of viable microorganisms treated with UVC LED were counted and compared with those of viable microorganisms not treated with UVC LED as controls. Tukey's Honestly Significant Difference was used to determine statistical significances (0.05). Units emitting UVC LED at 265 nm (12 mW), 265 nm (22.5 mW), and 280 nm (8 mW) killed *Candida albicans, Staphylococcus aureus,* methicillin-resistant *S. aureus* (MRSA), *E. faecalis, and Streptococcus sanguinis* after 30-90 seconds of exposure in laboratory assays (*p* < 0.05). Microbial killing differed among treatment times, UVC LED wavelengths, power levels of each unit, and specific microorganism. The unit emitting UVC LED at 265 nm (22.5 mW) killed *C. albicans, S. aureus,* MRSA, and *E. faecalis* in 30 s in root canals of extracted, instrumented teeth (*p* < 0.05). This dataset can be reused to assess the ability of other wavelengths and power levels to kill microorganisms as well as improve procedures for treating endodontic infections and inflammation in root canals.

**Specifications Table**

**Table:** 

Subject	Dentistry, Oral Surgery, and Medicine
Specific subject area	Ultraviolet C (UVC) light-emitting diode (LED) killing of microorganisms associated with endodontic and root canal infections
Type of data	Table and Figures
How the data were acquired	Optical power and energy meter (PM100D, Thorlabs, Inc., Newton, NJ 07860, USA) Photodiode Sensor (S120VC, 200-1100 nm, 50 mW; Thorlabs, Inc., Newton, NJ 07860, USA) Units emitting UVC LED at 265 nm (12 mW), 265 nm (22.5 mW), and 280 nm (8 mW) Microorganisms were treated with UVC LED emissions and were placed on agar-based microbiological media and incubated for 24 hours at 37°C
Data format	Raw and analyzed
Description of data collection	Energy levels of UVC LED units were measured using the PM100D meter with the S120VC, 200-1100 nm, 50 mW photodiode sensor Colony forming units (CFU) of viable microorganisms treated with UVC LED were counted and compared with the CFU of viable microorganisms not treated with UVC LED as controls
Data source location	Institution: University of Iowa College of Dentistry City: Iowa City Country: USA Location: 41.6628 (41°39′46″N), −91.5511 (91°33′4″W)
Data accessibility	Repository name: *Mendeley Data* DOI.10.17632/ck9ckzpfr7.3 Direct URL to data: https://data.mendeley.com/datasets/ck9ckzpfr7/3
Related research article	K. Morio, E. L. Thayer, A. M. Bates, K. A. Brogden, 255 nm LED kills *Enterococcus faecalis* and induces the production of cellular biomarkers in HEPM cells and gingival fibroblasts. *J. Endod*. 45 (2019) 774-783. https://doi.org/10.1016/j.joen.2019.02.016

**Value of the Data**

• This dataset documents the potent and lethal effects of UV LED for 5 oral microorganisms at 2 wavelengths and 3 power levels: 265 nm (12 mW), 265 nm (22.5 mW), and 280 nm (8 mW).
• The lethal effects of UV LED were rapid and broad-spectrum and killed oral microorganisms at 30-90 seconds *in vitro* in laboratory studies and at 30 seconds *ex vivo* in root canals of extracted, instrumented teeth.
• This approach has potential as a future treatment modality for clinicians treating caries, periodontal diseases, peri-implant diseases, and endodontic infections and inflammation in root canals.
• In future work, the synergistic interaction of UVC LED with sodium hypochlorite (NaOCl) could show improved microbial killing while using lower and less irritating concentrations of NaOCl.
• An approach using UVC LED for treatment of oral infections would reduce the use of antibiotics that are commonly prescribed in clinical practice and that contribute to the emergence of antimicrobial resistance.

## 1 Data Description

Endodontic infections in root canals are caused by *Eubacteria*, A*rchaebacteria*, and fungi [2], [3], [4], [5], [6]. Primary root canal infections often contain anaerobic gram-negative bacterial species and secondary, persistent root canal infections often contain facultatively anaerobic bacterial species [2,4]. Successful treatment of endodontic infections requires chemomechanical debridement of the canal spaces and proper sealing of the coronal and apical canal openings. Sodium hypochlorite (NaOCl) is often used to dissolve necrotic pulp tissue and disinfect the root canal. However, incomplete irrigation with NaOCl can lead to persist reinfections and chronic inflammation [2,7] and induce local pain and tissue damage if extruded [2,^8^,9].

Chemomechanical debridement is partially effective and UVC LED has been proposed as an alternative or adjunct for the disinfection process [1,7]. Recenty, we found that 255 nm UVC LED treatment killed *E. faecalis,* induced the production of cellular biomarkers in human embryonic palatal mesenchymal cells and gingival fibroblasts, and worked in synergy with NaOCl to more efficiently kill *E. faecalis* [1]. However, a treatment plan to use UVC LED as an alternative to or an adjunct with NaOCl would be have to be determined by the attending clinician and depend upon the clinical severity of the endodontic infection and inflammation.

Here, we extend our previous work with *E. faecalis* and show that units emitting UVC LED at 265 nm (12 mW), 265 nm (22.5 mW), and 280 nm (8 mW) wavelenths not only killed *E. faecalis* but additional microorganisms such as *C. albicans, S. aureus,* MRSA, and *S. sanguinis* on discs in laboratory assays. We also show that the unit emitting UVC LED at 265 nm (22.5 mW) wavelength killed *C. albicans, S. aureus,* MRSA, and *E. faecalis* in 30 seconds in the root canals of extracted, instrumented teeth.

UV LED is an attractive and practical approach to treating endodontic infections and inflammation in root canals [1,^7^,10]. UV light is known to alter DNA structure and DNA enzyme function [11]. Lethality of UV LED for microbial cells versus host tissues and cells is directly related to the size difference of these cells and the size of the UV LED wavelength used. Shorter wavelengths of UVC LED likely do not reach the DNA in the nucleus of host cells, which are >10 µm in size. The UVC LED is strongly absorbed by proteins and other molecules in the host cell cytoplasm [12]. Shorter wavelengths of UVC LED do reach the DNA in the cytoplasm of microbial cells, which are generally 0.5-2.0 µm in size [13]. Here UVC LED penetrates the microbial cell and is directly absorbed by the DNA forming cyclobutyl pyrimidine dimers (CPD) between adjacent thymines in the polynucleotide chains [14,15]. These CPDs then disrupt DNA transcription, translation, and replication leading to procaryotic cell death [11].

### 1.1 Characteristics of UVC LEDs

The energy levels of UVC LED units were measured with and without 1.0 mm fiberoptic (FO) filaments using the PM100D meter with the S120VC, 200-1100 nm, 50 mW photodiode sensor. The raw unanalyzed meter readings are in *Mendeley Data* Excel Table 1 (https://data.mendeley.com/datasets/ck9ckzpfr7/3). The analyzed data is in Table 1.

**Table 1:** Power and energy levels of 3 UVC LED units without and with fiberoptic (FO) filaments.

Unit parameters	UVC LED without FO filament	UVC LED with FO filament
**265 nm (12 mW)**^a^		
Diameter of unit hub or FO filament (mm)^b^	3.0	1.0
Range (min. to max.) (µW)	81.4-94.4	15.8-17.2
Mean value (Std. Dev.) (µW)	87.3 (3.0)	16.6 (0.4)
Time (seconds)	30	30
Energy dose (mJ)^c^	2.62	0.50
**265 nm (22.5 mW)**^a^		
Diameter of unit hub or FO filament (mm)^b^	3.0	1.0
Range (min. to max.) (µW)	166.3-190.9	1.6-11.4
Mean value (Std. Dev.) (µW)	151.0 (6.4)	10.9 (1.1)
Time (seconds)	30	30
Energy dose (mJ)^c^	4.53	0.33
**280 nm (8 mW)**^a^		
Diameter of unit hub or FO filament (mm)^b^	3.0	1.0
Range (min. to max.) (µW)	47.3-55.6	20.2-22.3
Mean value (Std. Dev.) (µW)	50.9 (1.7)	21.7 (0.6)
Time (seconds)	30	30
Energy dose (mJ)^c^	1.53	0.65

a Power readings (µW) for the 265 nm (12 mW) and 265 nm (22.5 mW) LED units were determined at 265 nm (n = 102 power meter readings). Power readings for the 280 nm (8 mW) LED unit was determined at 285 nm (n = 103 power meter readings). Power readings were determined using an energy meter (PM100D, Thorlabs, Inc., Newton, NJ 07860, USA) with a standard photodiode sensor (S120VC, 200 – 1100 nm, 50 mW (Thorlabs, Newton, NJ).

b The diameter of the UVC LED unit hub was 3.0 mm and the diameter of the FO filament was 1.0 mm.

c The energy dose (mJ, millijoules) was calculated as the power readings (µW) x time (seconds).

Without the 1.0 mm FO filament, the 265 nm (12 mW) LED emitted an average of 87.3 ± 3.0 (std. dev.) µW of power (Table 1). With the FO filament, this LED emitted an average of 16.6 ± 0.4 µW of power. This represented an 81.0% decrease. After 30 seconds, the energy doses were calculated to be 2.62 mJ and 0.50 mJ, respectively.

Without the filament, the 265 nm (22.5 mW) LED emitted an average of 151.0 ± 6.4 (std. dev.) µW of power (Table 1). With the FO filament, this LED emitted an average of 10.9 ± 1.1 µW of power and this represented a 92.8% decrease. After 30 seconds, the energy doses were calculated to be 4.53 mJ and 0.33 mJ, respectively.

Without the filament, the 280 nm (8 mW) LED emitted an average of 50.9 ± 1.7 (std. dev.) µW of power (Table 1). With the FO filament, this LED emitted an average of 21.7 ± 0.6 µW of power. This represented a 57.4% decrease. After 30 seconds, the energy doses were calculated to be 1.53 mJ and 0.65 mJ, respectively.

### 1.2 Characteristics of instrumented tooth models

**Fig. 1:**
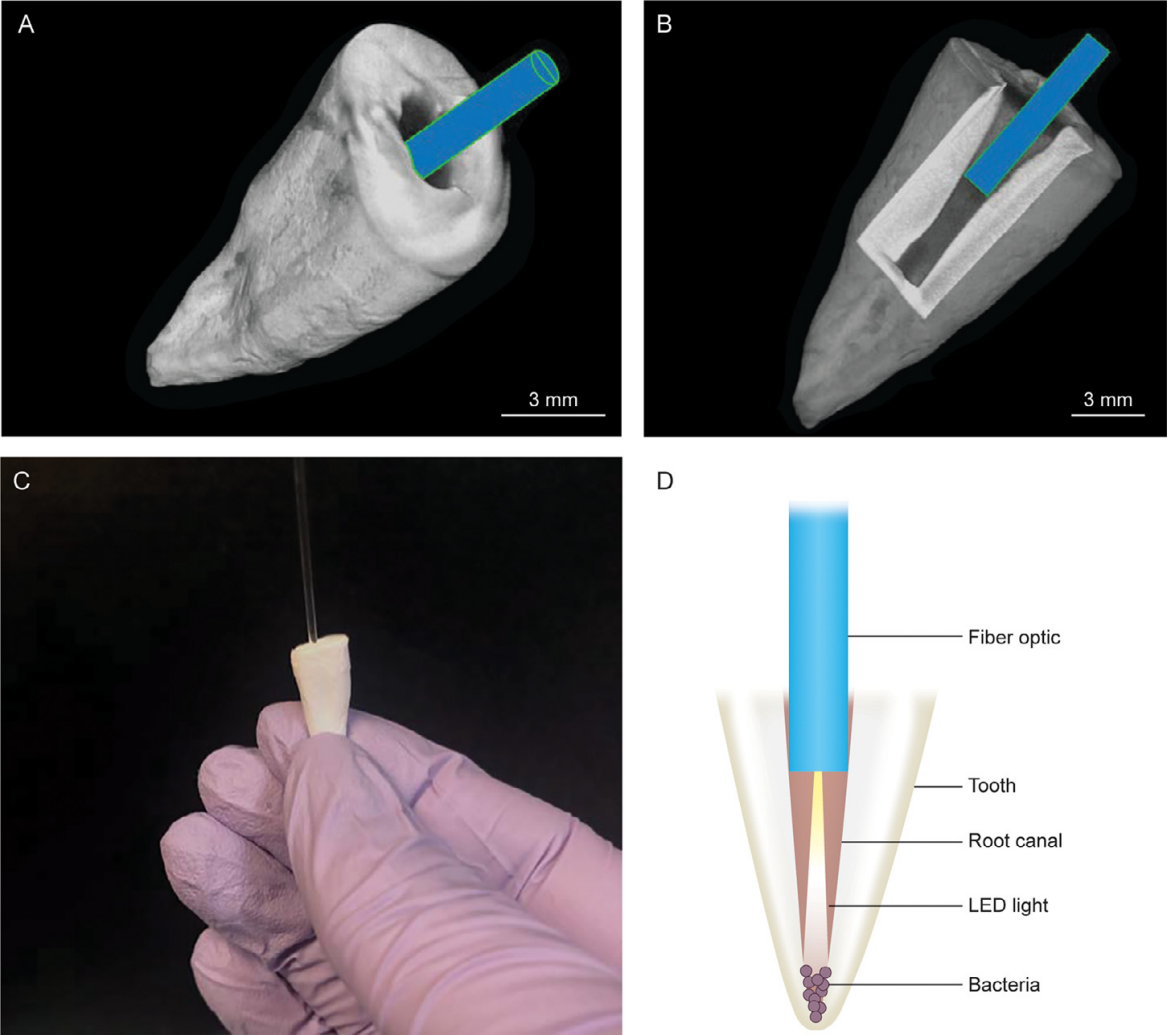
The instrumented model of root canals. Human premolar teeth were trimmed coronally to a uniform length of 15 mm and instrumented root canals were prepared, which allowed for the insertion of 1.0 mm fiberoptic (FO) filaments. (A) MicroCT image showing the external surface of the tooth with the FO filament entering from the top of the instrumented root canal. (B) MicroCT image of a cutaway section showing the canal containing the partially inserted FO filament. (C) The instrumented model of root canals containing bacteria being treated with UVC LED via the FO filament. (D) A schemata representation of the model showing the instrumented root canal, bacteria, and FO filament.

*Ex vivo* models of root canals were prepared from extracted single canal human premolars. MicroCT images showed the external surface of the tooth with the FO filament entering from the top of the instrumented canal (Fig. 1A). A cutaway section showed the partial insertion of the FO filament in the canal (Fig. 1B). Bacteria were added to this canal and treated with UVC LED via the 1.0 mm FO filament (Fig. 1C,D).

### 1.3 UVC LED killing of microorganisms on discs in laboratory assays

**Fig. 2:**
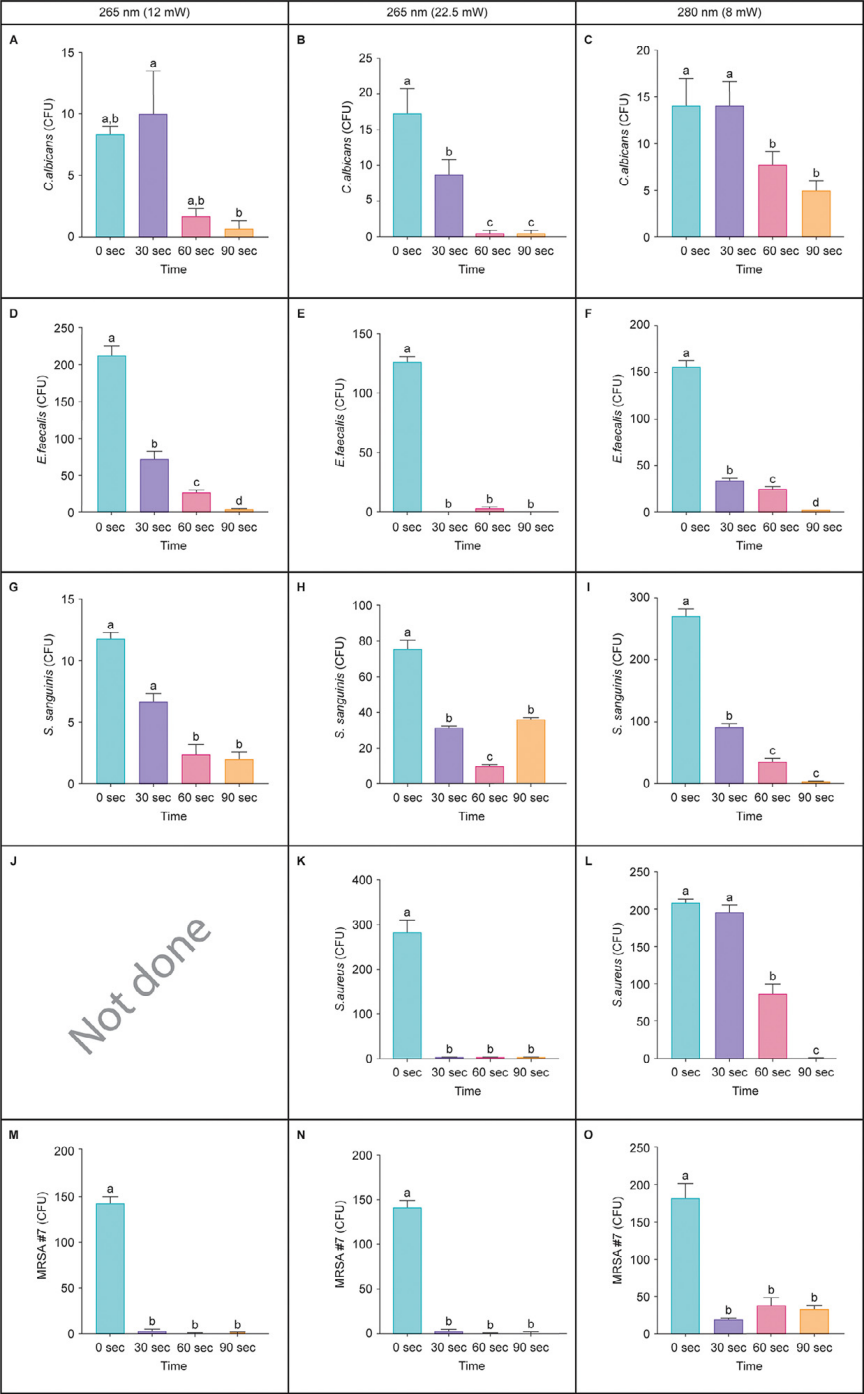
UVC LED killing of microorganisms on discs in laboratory assays. There were differences in time-induced killing of (A-C) *Candida albicans*, (D-F) *Enterococcus faecalis*, (G-I) *Streptococcus sanguinis*, (K, L) *Staphylococcus aureus*, and (M-O) methicillin-resistant *Staphylococcus* (MRSA #7) by UVC from (A, D, G, J, M) 265 nm from a 12 mW LED, (B, E, H, K, N) 265 nm from a 22.5 mW LED, and (C, F, I, L, O) 280 nm from a 8 mW LED. In graphs, bar values with the same letter(s) were not different. (*p* > 0.05).

There were differences in the killing of units emitting UVC LED at 265 nm (12 mW), 265 nm (22.5 mW), and 280 nm (8 mW) wavelenths for *Candida albicans, Staphylococcus aureus, MRSA, E. faecalis, and Streptococcus sanguinis* at 0-90 seconds on discs in laboratory assays. The raw unanalyzed CPU of microorganisms on discs in laboratory assays are in *Mendeley Data* Excel Table 2 (https://data.mendeley.com/datasets/ck9ckzpfr7/3). The analyzed data is presented in Fig. 2.

Microbial killng differed among treatment times, wavelength of UVC LED treatment, power level of each treatment, and the specific microorganism tested (Fig. 2). The 265 nm (12 mW) LED killed *E. faecalis* and MRSA at 30 seconds (*p* < 0.05; Fig. 2D,M) and killed *S. sanguinis* and *S. aureus* at 60 seconds (*p* < 0.05; Fig. 2G,J)*. C. albicans* was killed at 60 and 90 seconds (*p* > 0.05; Fig. 2A). The 265 nm (22.5 mW) LED killed *C. albicans, E. faecalis, S. sanguinis, S. aureus,* and MRSA at 30 seconds (*p* < 0.05; Fig. 2B,E,H,K,N). The 280 nm (8 mW) LED killed *E. faecalis, S. sanguinis,* and MRSA at 30 seconds (*p* < 0.05; Fig. 2F,I,O) and killed *C. albicans* and *S. aureus* at 60 seconds (*p* < 0.05; Fig. 2C,L).

### 1.4 UVC LED killing of microorganisms in root canals of extracted, instrumented teeth

**Fig. 3:**
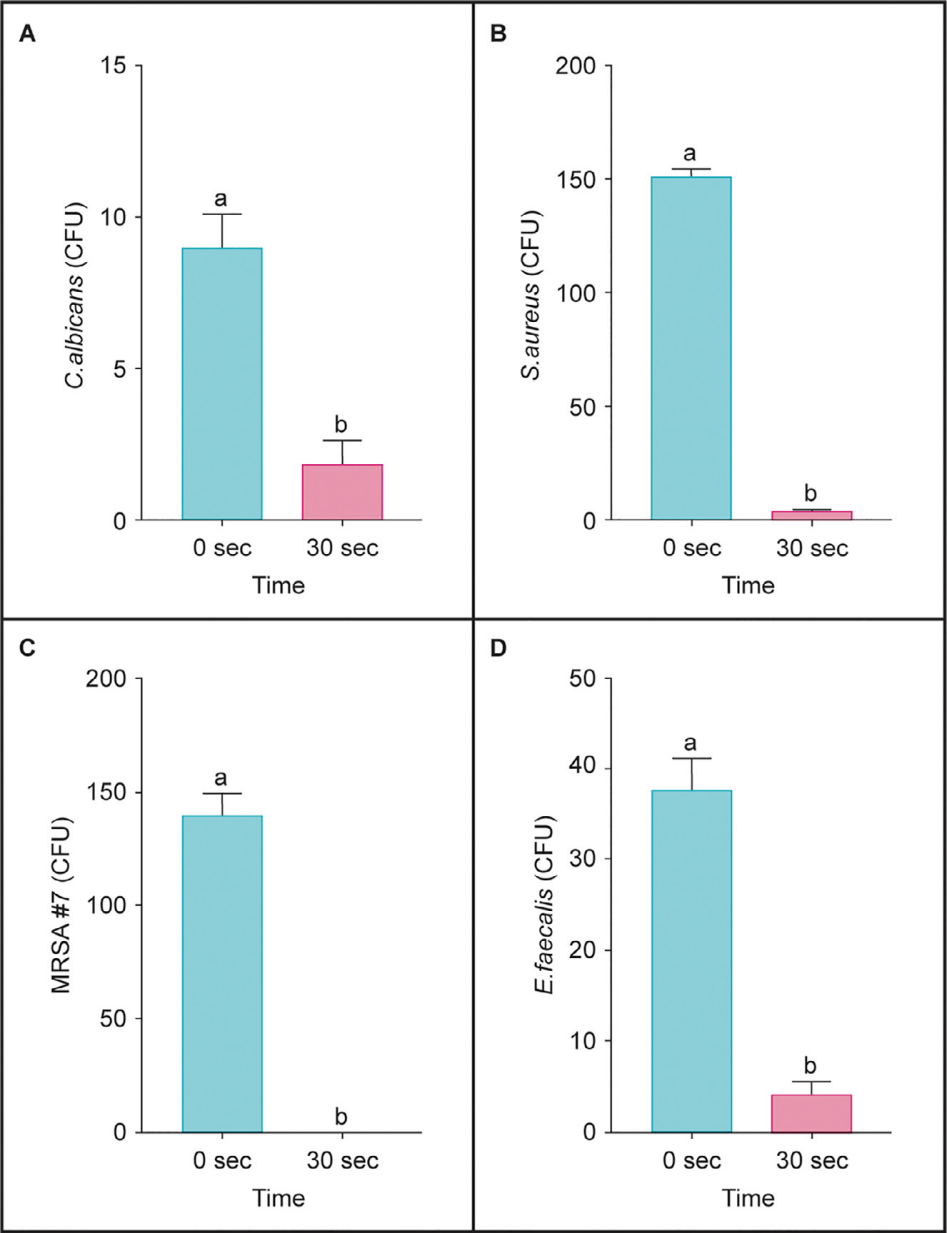
UVC LED killed (A) *Candida albicans,* (B) *Staphylococcus aureus,* (C) methicillin-resistant *Staphylococcus* (MRSA #7), and (D) *Enterococcus faecalis* in an instrumented tooth model of root canals. In graphs, bar values with the same letter(s) were not different. (*p* > 0.05).

The raw unanalyzed CFU of microorganisms in root canals of extracted, instrumented teeth are in *Mendeley Data* Excel Table 3 (https://data.mendeley.com/datasets/ck9ckzpfr7/3). The analyzed data is presented in Fig. 3.

The unit emitting UVC LED at 265 nm (22.5 mW) wavelength killed *C. albicans, S. aureus,* MRSA, and *E. faecalis* in 30 seconds in root canals of extracted, instrumented teeth (*p* < 0.05; Fig. 3).

## 2 Experimental Design, Materials and Methods

### 2.1 Microorganisms and culture conditions

C. *albicans* ATCC 64124, *E. faecalis* ATCC 29212, *S. sanguinis* ATCC 10556, and *S. aureus* ATCC 29213 were obtained from the American Type Culture Collection (ATCC, Manassas, VA). Methicillin-resistant *S. aureus* MRSA #7 was also used. All microorganisms were cultivated in BBL trypticase soy broth (Becton, Dickinson and Company, Sparks, MD) with 0.6% yeast extract (Janssen Pharmaceuticalaan, Geel, Belgium) and trypticase soy broth with yeast extract (TSBYE) containing Difco 1.5% agar (Becton, Dickinson and Company) at 37 °C.

Microorganisms were cultivated overnight on TSBYE agar, inoculated into TSBYE, and incubated at 37 °C. After 3 hours, the turbidity of culture suspensions was adjusted in TSBYE broth to an optical density of 0.108 at 600 nm (Spectronic 20D1; Thermo Fisher Scientific Inc, Waltham, MA). Plate counts were performed, and these suspensions contained 4.5-5.9 × 10^7^ colony-forming units (CFU) of each microorganism/ml.

### 2.2 Units emitting UVC LED

Three different UVC LED units were constructed*.* One unit emitted 265 nm from a 22.5 mW LED, one unit emitted 265 nm from a 12 mW LED, and one unit emitted 280 nm from an 8 mW LED. Units were fitted with 1.0 mm X 7.5 cm FO filaments. The diameter for the hub of the unit emitting UVC was 3.0 mm and the diameter of the FO filament was 1.0 mm.

Power levels emitted from the three units were measured without and with FO filaments using an optical power and energy meter (PM100D, Thorlabs, Inc., Newton, NJ 07860, USA) with a standard photodiode sensor (S120VC, 200-1100 nm, 50 mW; Thorlabs, Newton, NJ) for 30 seconds. The energy dose in millijoules (mJ) was calculated as the power readings of the meter (µW) x time of exposure (seconds).

### 2.3 UVC LED killing of microorganisms on discs in laboratory assays

UVC LED treatment of oral microorganisms was assessed as previously described for *E. faecalis* [1]. Briefly, 7.0 mm discs were punched from sterile cellulose nitrate filter membranes (#7182-002 membrane, 0.2 µm; Whatman International Ltd, Maidstone, UK) and 4 discs were placed onto blood agar plates containing trypticase soy agar with 5% defibrinated sheep blood (Remel, Lenexa, KS). 10 µl of culture containing 10^4^ CFU C. *albicans, E. faecalis, S. sanguinis, S. aureus*, or MRSA #7 was added to each disc. Each of the 4 discs were then treated with UVC LED for 0, 30, 60, and 90 seconds at a distance of 2 cm. After UVC LED treatment, the discs were placed into individual tubes containing 1 ml of TSBYE and vortex mixed to suspend the bacteria into solution. 50 µl from each tube was removed and spotted onto separate blood agar plates in triplicate. Plates were incubated overnight at 37 °C and CFU from each 50 µl spot were counted and recorded. This assay was repeated for each microorganism with each of the 3 units.

### 2.4 Preparation of root canals in extracted, instrumented teeth

The College of Dentistry maintains a large store of extracted teeth for educational and research purposes. These teeth are received in bulk regularly from dental clinicians throughout the state. Since the teeth do not have any associated clinical information or personal health information, the Institutional Review Board, Human Subjects Office at the University of Iowa has deemed that their use is not human subject research, and the Project does not have an IRB number.

Criteria for tooth selection included (i) freshly extracted single canal human premolar teeth, (ii) no previous history of root canal treatment, (iii) no root curvatures, (iv) no resorptive defects, and (v) no root fractures. Following extraction, premolars were cleaned of soft tissue and debris under biohazard safety protocols and teeth were immediately placed in sterile saline, autoclaved, and stored at 4 °C and 100% humidity.

Teeth were prepared as described by Oliveira et al. [16]. Decoronation was completed using a Brassler Diamond Disc (Brassler USA, Savannah, GA) on all teeth at the cementoenamel junction. All teeth were standardized coronally at a uniform length of 15 mm. The working length was determined 1 mm from standardized apex at 14 mm. Root canal systems were prepared using 0.02 tapered nickel titanium hand files (Dentsply, Tulsa, OK), varying tapered ProTaper (Dentsply, Tulsa, OK) S1 and S2 rotary files, and 0.04 tapered Endosequence File System (Brassler USA, Savannah, GA) to 40.04 with a final apical file of 50.02. Throughout instrumentation, each specimen was irrigated with 6 ml of 3% NaOCl followed by a final rinse of 3 ml of 17% EDTA for one minute to remove the smear layer during instrumentation. Following instrumentation, the specimens were placed in sterile saline and autoclaved.

Apical preparatory sealing and inoculation of the specimens was completed in the sterile laminar flow hood. Each specimen tooth was removed from the sterile saline solution and was allowed to air dry for two hours. Once dry, the exterior apex of each specimen was sealed with two coats of nail varnish and allowed to air dry for one hour. Sterile gauze held the specimens in place for tip placement and treatment.

### 2.5 Micro CT evaluation of root canals in extracted, instrumented teeth

Micro Computer Tomography was used to evaluate the FO filament tip placement in the root canal. A 1.0 mm × 7.5 cm FO filament was inserted into the instrumented root canal until resistance was felt (Fig. 1A,B). The tooth with the inserted FO filament was placed in a container, immobilized with expanded polystyrene packing, mounted on a brass base, and scanned using a Skyscan 1272 High Resolution Micro-CT (Bruker, Kontich, Belgium) at 80 kVp, 125 mA, 1500 millisecond exposure, 21 µm resolution with a 1 mm aluminum filter. NRecon software (Ver.1.7.1.0) was used to reconstruct the X-ray projections and CTVox (Ver.3.3.0 r1403) was used to assemble 3-D renderings of the premolars with the FO filament *in situ* (Fig. 1A,B). A longitudinal portion of the root was digitally removed to reveal the interior of the canal and extent of penetration of the FO filament. Due to the radiolucency of the FO filament within the canal, the boundaries of the filament were outlined based on the material density and a pseudo color was applied to the pixels within this density range.

### 2.6 UVC LED killing of microorganisms in root canals of extracted, instrumented teeth

10 µl of diluted culture containing 10^4^ CFU of *C. albicans, S. aureus,* MRSA, or *E. faecalis* was added to the instrumented root canal (Fig. 1C,D). The canal was treated with the 265 nm, 20–25 mW LED delivered via the FO filament for 30 seconds. An untreated control tooth received the same inocula. After treatment, channels from both teeth were rinsed with broth and concentrations of viable microorganisms were determined as described above.

### 2.7 Statistical analysis

CFU from each treatment were counted and recorded. One-way fixed-effect ANOVA models were fit to the control and UVC treated microbial CFU values. Pairwise group comparisons were conducted using the method of Tukey's Honestly Significant Difference (HSD). A 0.05 level was used to determine statistically significant differences. In Figs. 2 and 3 graphs, bar values with the same letter(s) were not different (*p* > 0.05). All analyzes were conducted using JMP (Version 10.0, SAS, Cary, NC).

## Ethics Statement

The College of Dentistry maintains a large store of extracted teeth for educational and research purposes. These teeth are received in bulk regularly from dental clinicians throughout the state. Since the teeth do not have any associated clinical information or personal health information, the Institutional Review Board, Human Subjects Office at the University of Iowa has deemed that their use is not human subject research, and the Project does not have an IRB number.

## CRediT authorship contribution statement

**Kimberly A. Morio:** Conceptualization, Methodology, Writing – review & editing. **Robert H. Sternowski:** Conceptualization, Methodology, Writing – review & editing. **Kim A. Brogden:** Conceptualization, Methodology, Investigation, Data curation, Writing – original draft, Writing – review & editing.

## Declaration of Competing Interest

All authors do not have financial affiliation (e.g., employment, direct payment, stock holdings, retainers, consultantships, patent licensing arrangements, or honoraria), or involvement with any commercial organization with direct financial interest in the subject or materials discussed in this manuscript, nor have any such arrangements existed in the past 3 years. Robert Sternowski is the president of Softronics, Ltd., Kim A. Brogden is an Emeritus Professor at the University of Iowa, and Kimberly Morio is an endodontist at Apex Endodontics. Robert H. Sternowski, Kim A. Brogden, and Kimberly Morio have patents related to the development of ultraviolet light-based technologies for clinical development to kill microorganisms in oral cavity infections.
